# A Case of Sepsis in Which Escherichia coli and Fastidiosipila sanguinis Were Isolated and Identified From a Blood Culture

**DOI:** 10.7759/cureus.66159

**Published:** 2024-08-05

**Authors:** Hisafumi Kihara, Haruka Yamamoto, Hisaharu Shikata, Masahiko Kaneko

**Affiliations:** 1 Department of Epidemiology and Public Health, Ehime University Graduate School of Medicine, Toon, JPN; 2 Department of Internal Medicine, Saiseikai Imabari Hospital, Imabari, JPN; 3 Department of Internal Medicine, Uwajima City Hospital, Uwajima, JPN

**Keywords:** urinary tract infection, blood culture, antimicrobial susceptibility, coinfection, fastidiosipila sanguinis

## Abstract

We isolated *Fastidiosipila sanguinis* for the first time in Asia, alongside *Escherichia coli*, from blood culture specimens in a case of complicated urinary tract infection with sepsis. In our case, *F. sanguinis* took 96 hours to form colonies under anaerobic culture and showed sensitivity to ceftriaxone, administered for the urinary tract infection. The pathogenicity and clinical significance of *F. sanguinis*, as well as its impact on the host when coinfected with other pathogens, require further analysis through the accumulation of cases.

## Introduction

We report a case of sepsis caused by a urinary tract infection in which *Escherichia coli* and *Fastidiosipila sanguinis* were detected and identified from a blood culture. *F. sanguinis* is a gram-positive anaerobic coccus that was first reported globally in 2005 and is considered a close relative of the genus *Clostridium* [1]. To the best of our knowledge, only two cases of *F. sanguinis* infection have been reported worldwide, and much remains unknown about its pathogenicity, retention rate, and reservoir in humans. Clinical information on rarely detected bacteria such as *F. sanguinis* is valuable and may provide crucial insights into the relationship between the in vivo microbiota and bacterial infections, including sepsis [2]. This report presents the clinical information and the process of detecting *F. sanguinis*.

## Case presentation

A 79-year-old woman had been experiencing fever and vomiting for the past two days and was taken to the emergency room after she began to exhibit impaired consciousness. She resided in a nursing home due to complications from cerebral infarction and had a urinary catheter inserted because of urinary dysfunction. Upon arrival at the emergency room, her level of consciousness was assessed as seven out of 15 points on the Glasgow Coma Scale (E4 V2 M1). Her blood pressure was 67/46 mmHg, heart rate was 128 beats per minute, body temperature was 40.0°C, oxygen saturation was 89% under ambient air, and respiratory rate was 32 breaths per minute. Adventitious sounds were heard in both lungs, and the abdomen was soft, but the lower abdomen was distended. We confirmed by visual inspection that the indwelling urinary catheter was obstructed midway, and the urine appearance was yellowish-white and thickly turbid.

**Figure 1:**
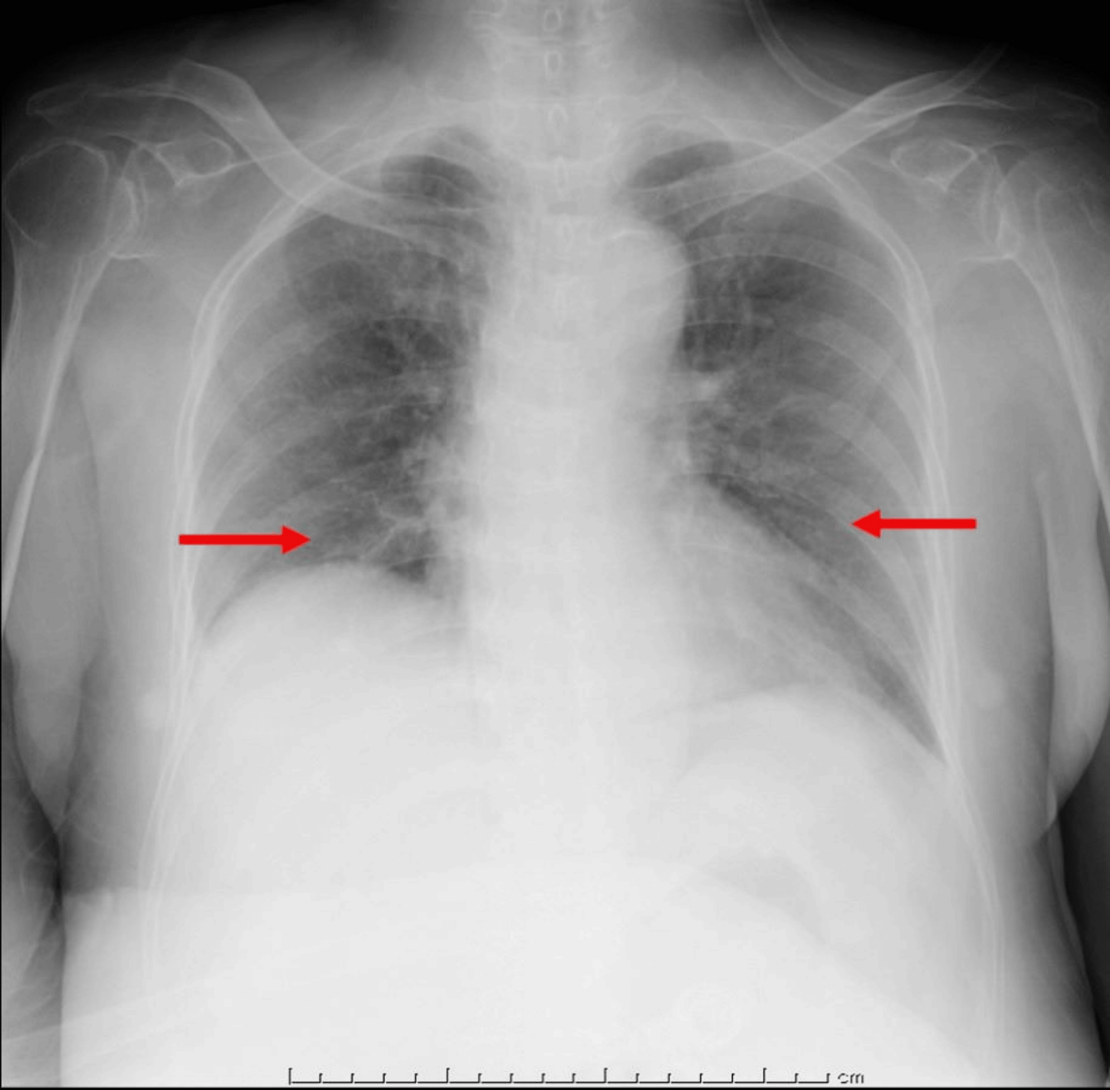
Chest X-ray The anterior-posterior X-ray view of the chest. The arrows indicate the ground glass shadows of both lung fields.

**Figure 2:**
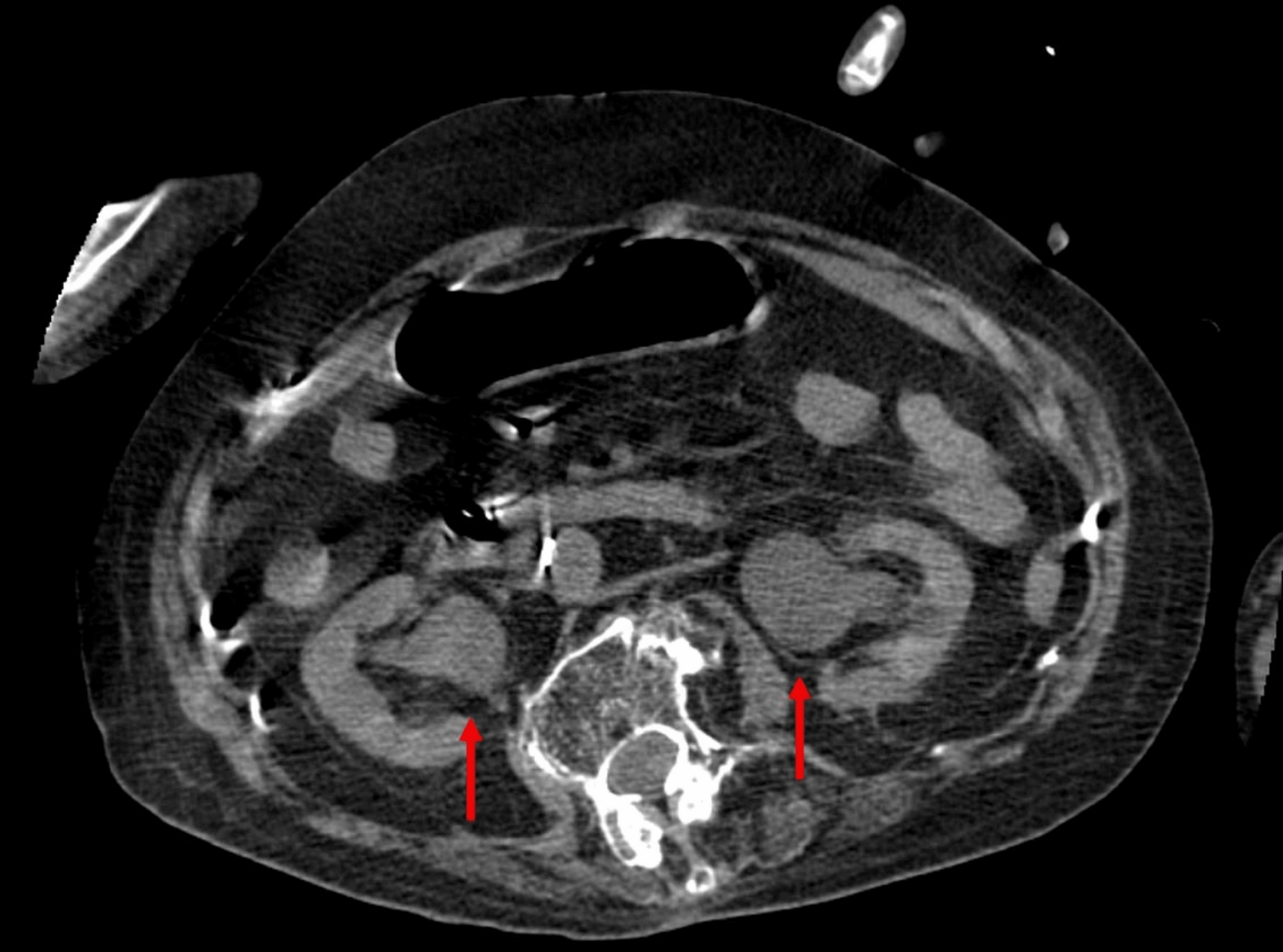
Abdominal CT Hydronephrosis was present in both kidneys. The arrows indicate an enlarged renal pelvis in both kidneys.

The white blood cell count was 14,600/µL (reference range: 3,500 - 9,000/µL), platelet count was 11.1 × 104/µL (13 - 36.9 × 10^4^/µL), serum creatinine was 1.79 mg/dL (0.6 - 1.1 mg/dL), C-reactive protein was 28.4 mg/dL (0.0 - 0.2 mg/dL), and procalcitonin was 1.52 ng/mL (< 0.5 ng/mL). Urinalysis revealed pyuria with positive nitrites and ketones. Imaging studies indicated signs of pneumonia suspected to be caused by aspiration of vomited material (Figure 1), as well as hydronephrosis and inflammatory changes in both kidneys (Figure 2). Based on these findings and the international consensus definitions for sepsis [3], the patient was diagnosed with sepsis due to urinary tract infection and aspiration pneumonia. 

She was promptly admitted to the intensive care unit for respiratory and circulation management. Urine and two sets of blood cultures were obtained and ceftriaxone (2 g/day) was administered. On the second day of culture, *E. coli* was detected in both blood and urine samples, indicating it as the causative bacteria for this infection. On the fifth day of culture, gram-positive cocci were detected in one of the aerobically cultured blood samples, but the specific bacterial species could not be identified using the matrix-assisted laser desorption/ionization time-of-flight mass spectrometry (MALDI-TOF MS) system. The unidentified gram-positive cocci (sample strain) were collected through pure culture, but the bacteria did not grow sufficiently to allow biochemical testing to identify the species. Therefore, the samples were sent to a specialized laboratory for identification testing.

**Figure 3:**
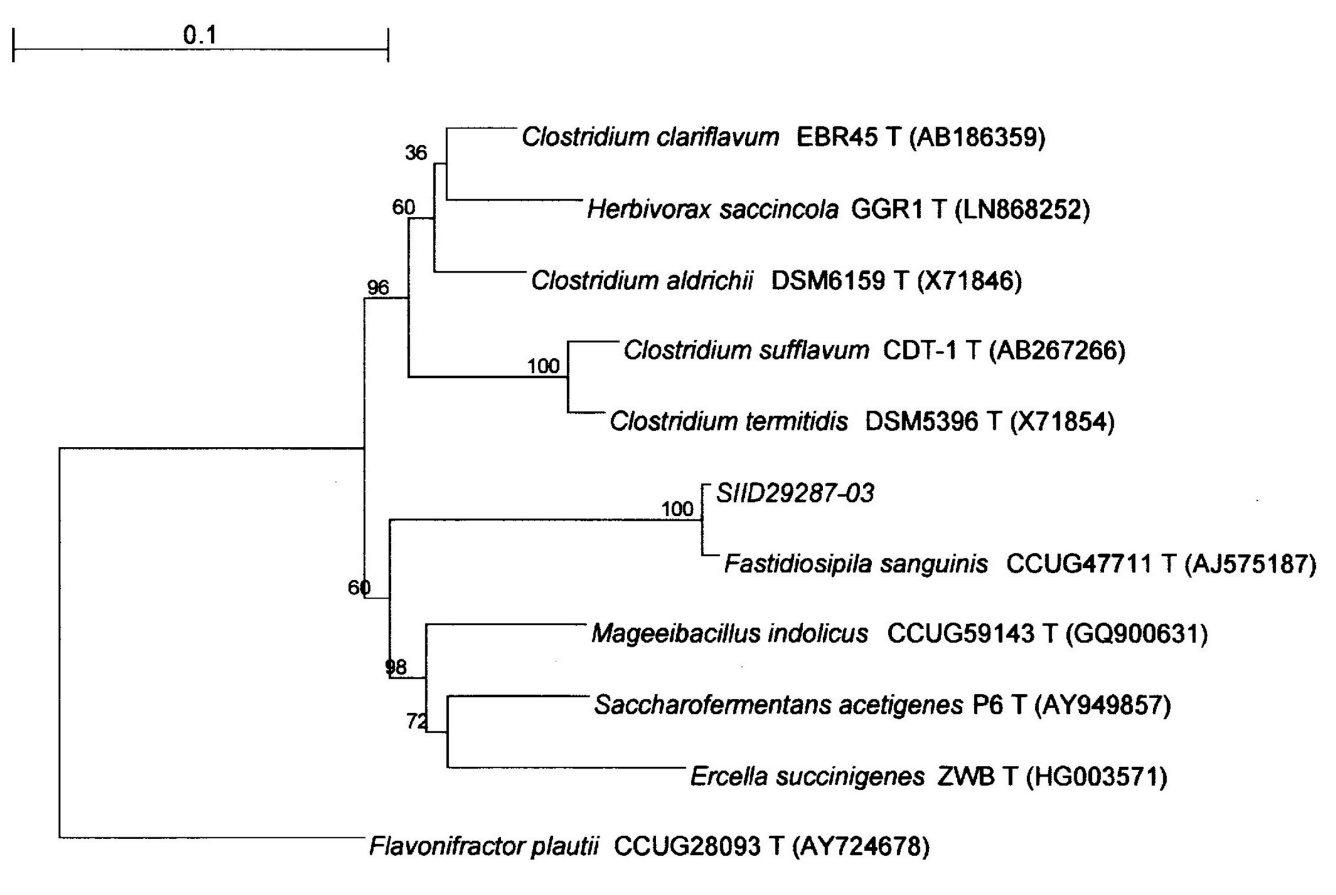
Molecular phylogenetic tree based on 16S rRNA gene type strain sequences The scale in the upper left corner, labeled 0.1, indicates the distance of 10% sequence divergence. The numbers written at each node of the molecular phylogenetic tree indicate the bootstrap probability (%), which represents the accuracy of the internal branches. Strain SIID29287-03 showed 100% bootstrap probability and was identified as *Fastidiosipila sanguinis.*

**Figure 4:**
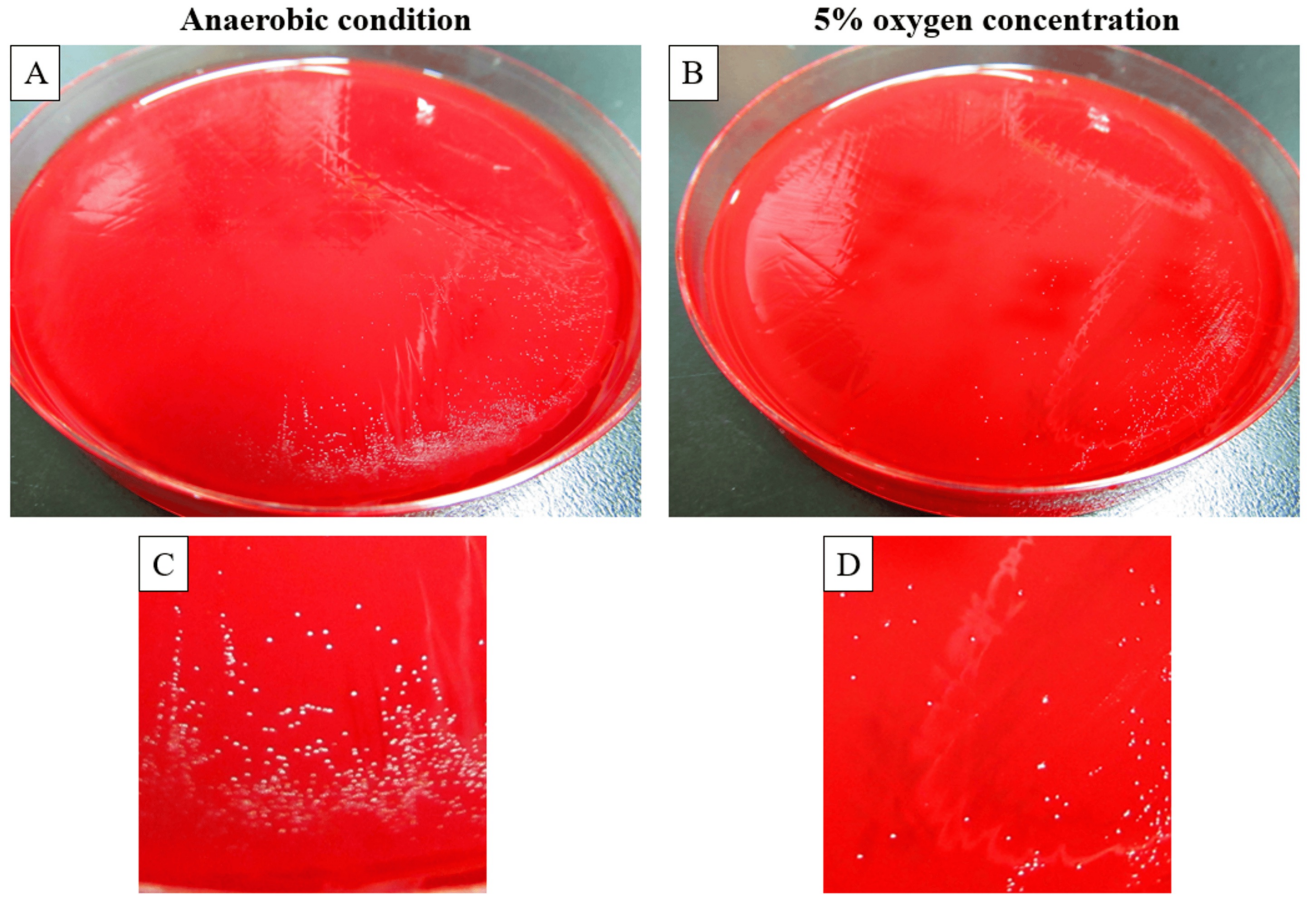
Incubation of the sample strain under anaerobic conditions and 5% oxygen concentration The sample strain was observed to form colonies under anaerobic conditions and 5% oxygen concentration after 96 hours of cultivation. (A) and (B) show the appearance of colonies formed on blood agar plates in anaerobically cultured and in the presence of 5% oxygen, respectively. (C) and (D) are enlarged views of portions of (A) and (B), respectively.

Analysis of the 16S rRNA gene and molecular phylogenetic tree revealed that the sample strain (sample ID: SIID29287-03) exhibited 100% sequence similarity with GenBank *F. sanguinis* CCUG 47711, confirming its identification as *F. sanguinis* (Figure 3). Sensitivity testing for the sample strain indicated susceptibility to benzylpenicillin, amoxicillin, vancomycin, imipenem, rifampin, and clindamycin, and resistance to metronidazole, levofloxacin, and ciprofloxacin, in accordance with the guidelines of the Clinical and Laboratory Standards Institute. The sample strain was cultured under both anaerobic and aerobic conditions with varying oxygen concentrations. Growth of the sample strain was confirmed under anaerobic conditions and at 5% oxygen concentration (Figure 4). It took approximately 96 hours for the sample strain to form colonies. 

Following treatment with ceftriaxone (2 g/day × 16 days) and comprehensive management, the patient showed improvement. On the 21^st^ day of hospitalization, the patient was discharged to a care facility.

## Discussion

We encountered a case of sepsis caused by a complicated urinary tract infection in which *E. coli* and *F. sanguinis* were identified through blood culture. Based on the bacterial load in the urine and detection in two sets of blood cultures, *E. coli* was considered the main causative pathogen of the urinary tract infection. Table 1 shows an overview of the two cases in which* F. sanguinis* has been detected to date and our case. 

**Table 1:** Comparison of three cases of infectious diseases The table shows clinical information on the three cases, the specimens in which *Fastidiosipila* *sanguinis* was detected, the culture conditions, and the incubation time until colony formation. Case 1 was cited from reference [4] and case 2 from reference [5].

Case	Age (years)	Sex	Type of infection	Main causative bacteria	Antibiotics used for treatment	Type of specimens	Oxygen conditions during incubation	Time to colony formation
1	84	Female	Osteitis	*Fastidiosipila* *sanguinis*	Amoxicillin	Capsular tissue Cortical bone Synovial fluid	Anaerobic and 5% carbon dioxide enriched	96 hours
2	Unknown	Male	Urinary tract infection	Moryella indoligenes	Piperacillin/tazobactam Metronidazole	Blood	Anaerobic	5 days
Present	79	Female	Urinary tract infection	Escherichia coli	Ceftriaxone	Blood	Anaerobic and 5% oxygen concentration	96 hours

The first recorded human case of *F. sanguinis* infection occurred in 2014, involving osteitis of the left toe in an 84-year-old female patient with type 2 diabetes [4]. *F. sanguinis* was detected in anaerobic and 5% carbon dioxide-enrich cultures of specimens from capsular tissue, cortical bone, and synovial fluid. The second case involved a urinary tract infection in an elderly man (age unknown) in which *F. sanguinis* was detected in an anaerobic blood culture bottle [5]. Evaluating the pathogenicity of *F. sanguinis* is challenging, but as demonstrated in the osteitis case, it can cause infectious disease independently. In the current case and others, it typically takes about four to five days from the start of culture for colonies to form. Therefore, the growth rate of *F. sanguinis* is relatively slow compared to that of major pathogens, which may hinder its ability to establish itself in infected tissue. Generally, blood culture tests are conducted for four to seven days to identify the causative bacteria [6]. However, the presence of slow-growing bacteria like *F. sanguinis* may be overshadowed by more dominant pathogens, potentially leading to an underestimation of its frequency.

From a different perspective on pathogenicity, it is increasingly recognized that interactions between the causative pathogen and other bacteria from the microbiome can impact the clinical course of the host [7]. For example, coinfection involving *Pseudomonas aeruginosa* and other pathogenic bacteria can enhance the persistence of infectious diseases [8]. It has been suggested that polymicrobial infections caused by certain gram-positive cocci may influence the progression of urinary tract infections [9].

In our case, although *F. sanguinis* was detected in only one set of blood culture specimens, like other gram-positive cocci, it may occur as a coinfection in complicated urinary tract infections. *F. sanguinis* might also influence virulence through coinfection with other pathogenic bacteria. Indeed, the presence of the genus *Fastidiosipila* in the microbiota has been linked to premature birth [10] and high-risk human papillomavirus infection [11].

Regarding antibiotic treatment for *F. sanguinis*, based on the results of our antimicrobial susceptibility testing, the detected *F. sanguinis* showed sensitivity to penicillin, meropenem, and clindamycin, and resistance to metronidazole. These susceptibility results were consistent with those reported previously [4,5]. In our case, ceftriaxone (2 g/day × 16 days), which was initially used for empirical treatment of urinary tract infection, was also effective against *F. sanguinis*.

## Conclusions

We report the detection of *F. sanguinis*, along with *E. coli*, in blood culture specimens from a patient with a severe complicated urinary tract infection. *F. sanguinis* has been rarely reported because it requires four to five days of incubation under anaerobic conditions to form colonies. Our case represents the first detection of *F. sanguinis* in Asia. Identification of *F. sanguinis* was time-consuming, but treatment with ceftriaxone was successful without clinical complications. There have been few reports worldwide of human infections where *F. sanguinis* has been detected, and more cases need to be documented to clarify its pathological significance.
